# Deep Eutectic Solvent + Water System in Carbon Dioxide Absorption

**DOI:** 10.3390/molecules29153579

**Published:** 2024-07-29

**Authors:** Jing Fan, Xin Zhang, Nan He, Fenhong Song, Hongwei Qu

**Affiliations:** School of Energy and Power Engineering, Northeast Electric Power University, Jilin 132012, China; crystalfan@neepu.edu.cn (J.F.); 2202200471@neepu.edu.cn (X.Z.); henan@neepu.edu.cn (N.H.); fenhongsong@neepu.edu.cn (F.S.)

**Keywords:** deep eutectic solvent, CO_2_ capture, isovolumetric saturation theory, absorption mechanism

## Abstract

In the present work, deep eutectic solvents (DESs) were synthesized in a one-step process by heating the hydrogen bond acceptors (HBAs) tetrabutylammonium bromide and tetrabutylphosphonium bromide, along with two hydrogen bond donors (HBDs) ethanolamine and N-methyldiethanolamine, which were mixed in certain molar ratios. This mixture was then mixed with water to form a DES + water system. The densities of the prepared DES + water systems were successfully measured using the U-tube oscillation method under atmospheric pressure over a temperature range of 293.15–363.15 K. The CO_2_ trapping capacity of the DES + water systems was investigated using the isovolumetric saturation technique at pressures ranging from 0.1 MPa to 1 MPa and temperatures ranging from 303.15 K to 323.15 K. A semi-empirical model was employed to fit the experimental CO_2_ solubility data, and the deviations between the experimental and fitted values were calculated. At a temperature of 303.15 K and a pressure of 100 kPa, the CO_2_ solubilities in the DES + water systems of TBAB and MEA, with molar ratios of 1:8, 1:9, and 1:10, were measured to be 0.1430 g/g, 0.1479 g/g, and 0.1540 g/g, respectively. Finally, it was concluded that the DES + water systems had a superior CO_2_ capture capacity compared to the 30% aqueous monoethanolamine solution commonly used in industry, indicating the potential of DES + water systems for CO_2_ capture.

## 1. Introduction

The surge in carbon dioxide (CO_2_) emissions has led to a multitude of problems for human society, most notably the exacerbation of the global greenhouse effect. This phenomenon has triggered the melting of Arctic ice, contributing to rising sea levels and an increased incidence of natural disasters worldwide [1,2,3]. With regard to this issue, the primary strategies for mitigating CO_2_ emissions encompass the development of alternative energy sources, such as wind, solar, nuclear power, and combustible ice [4,5,6,7,8,9,10].

To further minimize emissions, advancements in coal combustion technology are crucial. These advancements aim to reduce emissions during the combustion process [11]. Moreover, the desulfurization and decarbonization of flue gases before their release into the atmosphere are crucial in curbing their environmental impacts. Among the most cost-effective and efficient approaches to reduce the harm to the environment are the enhancement of coal combustion processes and the implementation of post-combustion carbon capture from flue gases [12].

Deep eutectic solvents (DESs) have emerged as promising agents in the realm of gas absorption, particularly for CO_2_ capture, which is due to their remarkable solubility for CO_2_ [13,14,15,16,17]. Their potential application in this field underscores the need for continued research and development to harness their capabilities fully. DESs are emerging as effective agents for CO_2_ absorption, with mechanisms typically divided into physical and chemical absorption. In the realm of physical absorption, choline-based solvents, such as those prepared with choline chloride and triethylene glycol in a 1:4 molar ratio, have been found to exhibit exceptionally high CO_2_ solubility. This type of absorption generally follows the principles of gas solubility, where the solubility of CO_2_ increases with the increase in pressure and the decrease in temperature [18,19].

On the other hand, chemical absorption has been demonstrated to be more efficient than its physical counterpart. For instance, DESs with monoethanolamine as the hydrogen bond donor (HBD) engage in a chemical reaction with CO_2_ to form carbamates. This process not only enhances the absorption capacity but also mitigates the corrosive effects on the equipment, which are often observed when pure monoethanolamine solutions are used [20]. The reduced corrosiveness can be attributed to the network of hydrogen bonds formed by the HBD and hydrogen bond acceptor (HBA), which helps alleviate redox cycling and, consequently, lessens the corrosive impact on the instruments. However, DESs are characterized by a higher viscosity compared to conventional solvents, which can further increase during the gas absorption process. This increased viscosity may hinder the gas–liquid mass transfer, potentially affecting the overall efficiency of the absorption process [21,22,23,24,25]. Despite this, the unique properties of DESs, including their tunability and biodegradability, make them promising candidates for CO_2_ capture technologies.

In recent studies, the behavior of DESs in CO_2_ absorption has been extensively investigated. Zheng [26] discovered that alcoholic amine solvents in ethanol, such as triethylenetetramine and tetraethylenepentamine, formed white precipitates upon exposure to CO_2_, a phenomenon not observed in aqueous solutions of triethylenetetramine. Luo [27] explored the solubility of CO_2_ in a mixture of diethylenetriamine, cyclobutanesulfone, and water under different temperatures and pressures (up to 400 kPa). Meanwhile, Ali [28] examined various DESs with different HBAs containing phosphorus and amino groups, revealing that the CO_2_ capture capacity was influenced by the type of salt in the DES and the molar ratio of HBD to HBA. Wang [29] synthesized a series of DESs using tetrabutylphosphonium bromide as the HBA and phenol as the HBD, and evaluated their CO_2_ trapping efficiency at pressures of less than 2000 kPa. The results indicated that phosphorus-based DESs possess strong hydrogen bonding and exhibit superior CO_2_ trapping capabilities. Adeyemi [30] determined the effect of a 30% ethanolamine solution on CO_2_ absorption, and at atmospheric pressure, the absorption of CO_2_ by a DES was similar to that of the ethanolamine solution, at 0.12 g/g. Lee [31] prepared an imidazolium DES which absorbed CO_2_ up to 0.114 g/g. At 318 K and 5 MPa, Altamash [32] prepared betaine DES which could absorb up to 0.158 g/g of CO_2_.

With this backdrop, the current study synthesized twelve DESs using monoethanolamine (MEA) and N-methyldiethanolamine (MDEA) as HBDs, and tetrabutylammonium bromide (TBAB) and tetrabutylphosphonium bromide (TBPB) as HBAs. These DESs were prepared with the HBD to HBA molar ratios of 1:8, 1:9, and 1:10, and then mixed with water in a 1:1 mass ratio to form low-viscosity DES + water systems. The densities of these solvents and their CO_2_ solubilities were subsequently measured, providing further insights into the potential of these DESs for CO_2_ capture.

## 2. Results and Discussion

### 2.1. Density of DES + Water Systems

The density data were measured using an Anton Paar DMA 5000 M densitometer (Anton Paar GmbH, Graz, Austria), as presented in Tables S1 and S2 (see Supplementary Materials). In the present study, the densities of the prepared DES + water systems were correlated using a linear fitting method. The relevant parameters were derived from the correlation, given by Equation (1):

(1) ρ=A+BT

where *ρ* is the density, *T* is the temperature, and *A* and *B* are constants.

Specific parameters are displayed in Table 1.

**Figure 1:**
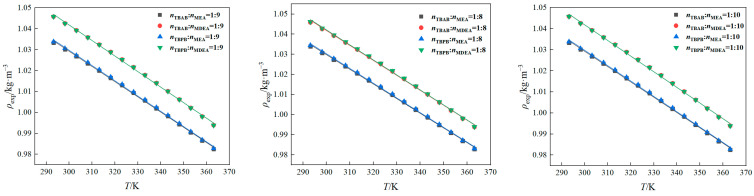
Experimental data for the densities of 50 wt% DESs.

Figure 1 shows the density data for the prepared DESs at various temperatures. It is observed that an increase in temperature leads to a decrease in density. This trend can be explained by the weakening of intermolecular hydrogen bonds with the increase in temperature. The weakening of intermolecular hydrogen bonds results in reduced intermolecular forces and an increase in molecular motion, which in turn causes the density to decrease [33]. Additionally, the thermal expansion of the DES + water systems increases the volume, which also contributes to this effect, thus resulting in a decrease in density at elevated temperatures.

Furthermore, the density of the system gradually decreases as the molar ratio of the alcohol-amine solution increases, which is due to the lower density of the alcohol-amine liquid itself. Notably, among various DES + water systems with the same type of HBD, those containing MDEA exhibited a higher density compared to those with MEA, which is due to the fact that pure MDEA has a greater density than pure MEA [34].

### 2.2. Solubility of CO_2_ in DES + Water Systems

**Figure 2:**
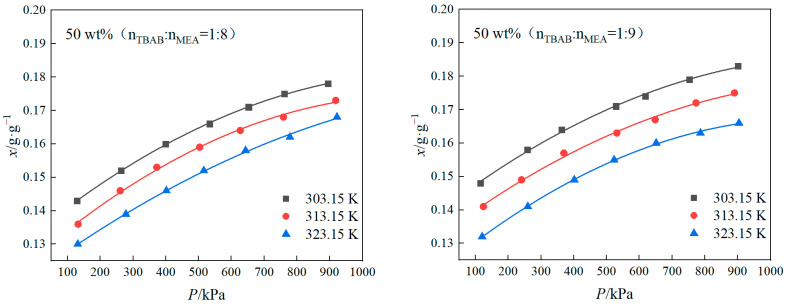

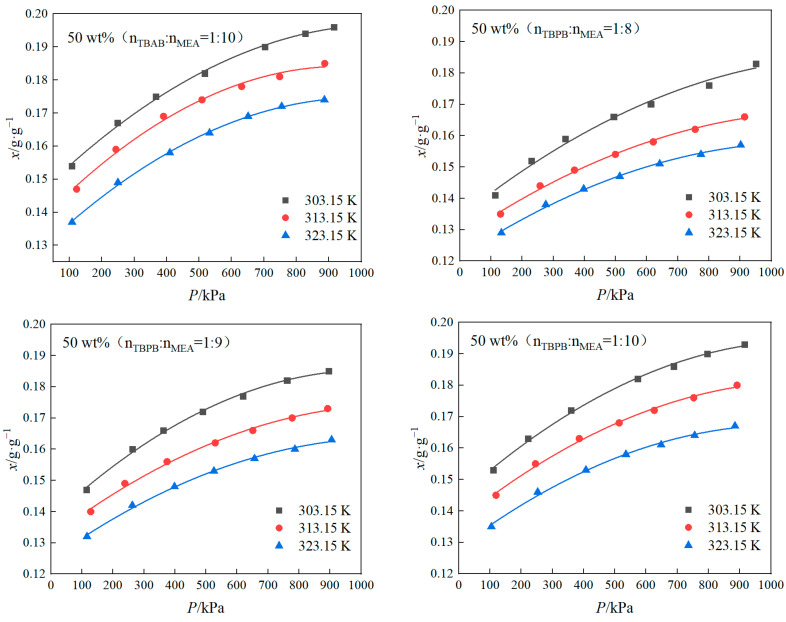
CO_2_ solubility curves of 50 wt% TBAB + MEA and 50 wt% TBPB + MEA eutectic solvents versus pressure.

**Figure 3:**
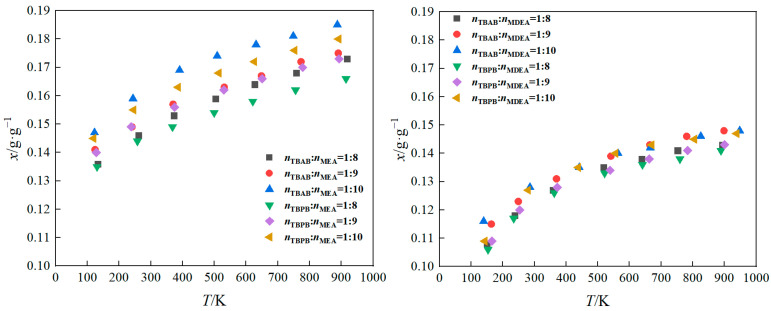
CO_2_ solubility curves at 313.15 K.

In the present study, the solubility of CO_2_ in a 50 wt% DES mixed with water was measured over a temperature range of 303.15–323.15 K and a pressure range of 0.1–1 MPa. A semi-empirical model was employed to correlate the solubility of CO_2_ with both the temperature and the pressure, with the resulting fitted curves depicted in Figure 2 and Figure 3. The semi-empirical model [35] is given by Equation (2):

(2) $$\ln P_{CO_{2}}=a+\frac{b}{T}+c\alpha +\frac{d\alpha }{T}+e\alpha^{2}$$

where *P_CO_*_2_ is the CO_2_ pressure in the diffusion chamber at dissolution equilibrium (kPa), *T* is the absorption temperature at equilibrium (K), and *α* is the molar solubility (mol/mol^−1^).

Table 2 lists the fitting parameters for various compositions of the mixtures, specifically for TBAB/MEA, TBAB/MDEA, and TBPB/MDEA. For the 50 wt% TBAB/MEA mixture, the average relative deviation is 0.54%. For the 50 wt% TBAB/MDEA mixture, the average relative deviation is 0.76%.

In the case of the 50 wt% TBPB/MDEA mixture, the average relative deviation is 0.51%. The results obtained for the 50 wt% TBPB/MEA mixture were the same as those for the TBPB/MDEA mixture with an average relative deviation of 0.51% but then “higher values of 0.87%”.

Based on Figure 2, as well as the data presented in Tables S3 and S4, it is evident that the HBD has a more significant impact on the solubility of CO_2_ than the HBA. When the amount of HBD was held constant, the solubility of CO_2_ in the DES followed a descending order: MEA > MDEA. This trend can be explained by the higher pH value of MEA (12.1) at 293.15 K compared to the pH of MDEA (11.5). Since CO_2_ is an acidic gas, its solubility in a DES + water system containing MEA is greater than that in a system containing MDEA.

Consequently, the CO_2_ solubility in the DES + water systems with MEA was higher than in those with MDEA. The saturation absorption of CO_2_ for the DES containing MEA consistently exceeded those containing MDEA. This is due to the direct reaction between CO_2_ and the primary amine group present in MEA, which leads to faster absorption and consequently higher solubility of CO_2_.

As a primary amine, MDEA is chemically stable and absorbs CO_2_ primarily through a hydrolysis reaction, which can result in relatively lower absorption rates compared to secondary amines. When a DES containing MDEA is mixed with water, the CO_2_ absorption in the system involves both physical and chemical processes. The process is explained by the alkaline catalytic principle, where MDEA acts as a catalyst for CO_2_ hydrolysis, interacting with the protons generated during the reaction to facilitate the absorption of CO_2_.

Increasing the temperature reduces the CO_2_ uptake of the DES + water systems at saturation, with all eutectic solvents showing decreased CO_2_ uptake at 313.15 K and 323.15 K. Based on Figure 3, the CO_2_ solubility in the DES + water systems increased with an increase in the molar ratio of the alcohol-amine solution. When the proportion of hydrogen bond donors in the DESs + water system is increased, the chances of CO_2_ gas coming into contact with the HBDs are greatly increased, which results in a much greater chance of chemical reaction between CO_2_ gas and the hydrogen bond donors, which in turn results in an increase in the solubility of CO_2_ [36].

According to the results presented in Tables S5 and S6, at a temperature of 303.15 K and a pressure of 100 kPa, the CO_2_ solubilities in the DES + water systems containing TBAB and MEA, with molar ratios of 1:8, 1:9, and 1:10, were 0.1430 g/g, 0.1479 g/g, and 0.1540 g/g, respectively. The corresponding CO_2_ solubilities based on the moles of MEA were 0.6514 mol/mol amine, 0.6498 mol/mol amine, and 0.6477 mol/mol amine, respectively. It is observed that increasing the molar ratio of the alcohol-amine solution enhances the overall CO_2_ solubility in the system, though this comes at the cost of reduced utilization efficiency of the alcohol-amine solution.

**Figure 4:**
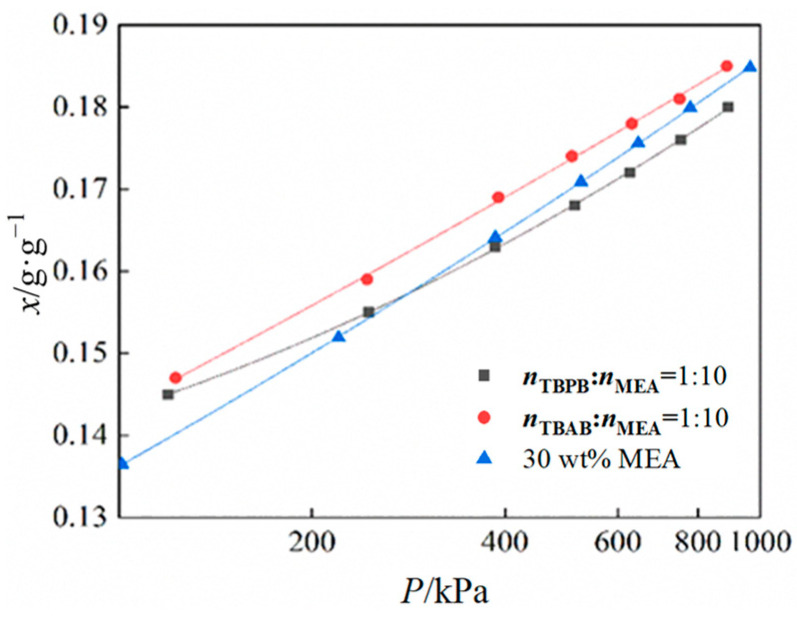
Comparison of the CO_2_ solubilities for 50 wt% TBAB + MEA, 50 wt% TBPB + MEA, and 30 wt% MEA under different pressures.

At a temperature of 313.15 K, the CO_2_ solubility of a DES + water system was compared with that of a 30 wt% aqueous MEA solution. As shown in Figure 4, when the MEA to HBA molar ratio was 1:10, the CO_2_ solubility of the TBAB + MEA system was higher than that of the 30 wt% MEA solution within the pressure range of 0.1–1 MPa.

However, as the pressure increased, the saturated solubility of the MEA solution surpassed that of the DES + water systems. This is attributed to the complex hydrogen bonding network formed by the DES and the HBA, which enhances the solubility of CO_2_ at lower pressures. The addition of water to the DES + water systems weakened this hydrogen bonding, leading to the formation of carbamates and carbonates as CO_2_ was absorbed. This reaction increased the solvent viscosity and, with a further increase in pressure, eventually leveled off the solvent’s CO_2_ uptake. Also, the CO_2_ solubility of the DES + water system synthesized in this work exceeds that already reported for imidazolyl DES (0.114 g/g) and betaine-based DES (0.158 g/g).

## 3. Mechanism of CO_2_ Absorption in DES + Water Systems

Acting as HBDs, MEA and MDEA chemically react with CO_2_ in the presence of water. More specifically, when TBAB was combined with MEA, the primary amine group in MEA reacted with CO_2_ to form a carbamate, thereby immobilizing the CO_2_.

In the DES + water systems, the reaction mechanism involved the interaction of CO_2_ with the amine group of MEA to produce a hydrophilic ammonium salt. The introduction of water moderated the hydrogen bonding within the solvent, which facilitated the reaction. Subsequently, the ammonium salt dissolved in water, forming a carbonate. The reaction mechanism can be summarized as follows [37]:

$$\mathrm{R-NH}+{\mathrm{CO}}_{2}\Leftrightarrow {\mathrm{R-NH}}^{+}{\mathrm{CO}}_{2}$$

$$\mathrm{R-NH}+{\mathrm{R-NH}}^{+}{\mathrm{CO}}_{2}\Leftrightarrow {{\mathrm{R-NH}}_{2}}^{+}{+{\mathrm{R-NCOO}}_{2}}^{-}$$

$${{\mathrm{R-NCOO}}_{2}}^{-}+H_{2}O\Leftrightarrow {{\mathrm{R-NH}}_{2}+{\mathrm{HCO}}_{3}}^{-}$$

## 4. Experimental

### 4.1. Reagents

In the present study, tetrabutylammonium bromide (TBAB) and tetrabutylphosphonium bromide (TBPB) were chosen as the HBAs. Monoethanolamine (MEA) and N-methyldiethanolamine (MDEA) were employed as HBDs. All reagents were provided by Shanghai Weili Co., Ltd., Shanghai, China, and used without additional purification. The specific characteristics of the reagents are presented in Table 3.

### 4.2. Preparation of DES + Water Systems

In these experiments, the DES + water systems were synthesized in a single step using a heating method. The HBAs used were TBAB and TBPB, while MEA and MDEA served as the HBDs. Water was employed as a mixing agent.

The HBDs, HBAs, and water were combined in a flat-bottom flask. The flask was then placed in a magnetic stirrer that was equipped with a heat-collecting system. The stirring was set at a temperature of 75 °C and a rotational speed of 800 revolutions per minute (r/min) for a duration of 1.5 h and continued until the mixture became clear and transparent.

The DES was cooled to room temperature, put into a vacuum drying oven to remove impurities, and observed for 24 h. If no crystallization occurred, it was considered that the preparation of DES was successful, and the prepared DESs were used as the base solutions to which 50% deionized water was added. Then, these samples were put into a magnetic stirrer, the temperature was set to room temperature, and stirring was performed for 30 min. The moisture content of DES + water systems was measured by the Metrohm Karl Fischer Titrator C30s (Metrohm, Herisau, Switzerland).

### 4.3. Density Measurements

The Anton Paar DMA 5000M Density Meter employs the U-tube oscillation technique for measuring the density of substances, which is currently recognized as one of the most accurate methods in the world. The equipment boasts an uncertainty of 0.0001 g/cm^3^ and an impressive repeatability of 0.000001 g·cm⁻^3^.

This technique operates on the principle that a magnet housed within a U-tube oscillates periodically. When a liquid is introduced, the resulting difference in density alters the oscillator’s natural frequency. This change in natural frequency subsequently affects the period of oscillation. The instrument maintains a constant internal temperature during the experiment, ensuring the accurate determination of density at various temperatures. This is achieved by compensating for changes in the liquid’s volume and mass within the U-tube that occur due to variations in temperature.

### 4.4. Isovolumetric Saturation Theory

The isovolumetric saturation theory is a precise method for measuring gas solubility [38]. The specific procedure is as follows: the gas and liquid to be measured are placed in the gas and diffusion chambers, respectively. After the pressure and temperature have stabilized, the diffusion chamber is charged with CO_2_. The solubility of the gas under the resulting pressure is then determined.

The solubility is calculated by Equation (3):

(3) $$\alpha =\frac{n_{p_{e}}}{n_{s}}$$

where *α* is the CO_2_ solubility of the absorbent (mol CO_2_/mol DES), *n*_s_ is the amount of absorbent (mol), and *n_Pe_* is the amount of CO_2_ absorbed by the DES (mol).

Before calculating *n_s_*, the average relative molecular mass of the absorbent is calculated, where the relative molecular mass of the DES is calculated by Equation (4):

(4) $$M_{DESs}=M_{HBA}\cdot x_{HBA}+M_{HBD}\cdot x_{HBD}$$

where *M_DESs_* is the average relative molecular mass of the *DES*, *M_HBA_* is the relative molecular mass of the *HBA*, *x_HBA_* is the mole fraction of the *HBA* in the *DES*, *M_HBD_* is the relative molecular mass of the *HBD*, and *x_HBD_* is the mole fraction of the *HBD* in the *DES*.

When a certain mass *w* of the *DES* is taken as the absorbent, the amount of substance *n_s_* is calculated by Equation (5):

(5) $$n_{s}=\frac{w}{M_{DESs}}$$

The amount of CO_2_ absorbed by *DESs*, *n_Pe_*, is the difference between the gas entering the diffusion chamber and the gas remaining in the diffusion chamber at the end of the absorption, which is calculated by Equation (6):

(6) $$n_{p_{e}}=\left[\rho_{g(P_{1})}v_{1}-\rho_{g(P_{3})}v_{1}-\rho_{g(P_{e})}(v_{2}-\frac{w}{P_{DESs}})\right]/M_{CO_{2}}$$

where *ρ_g_*_(*P*1)_ is the density of the gas in the gas chamber before the start of absorption (kg·m^−3^), *ρ_g_*_(*P*3)_ is the density of the gas in the gas chamber after absorption (kg·m^−3^), *ρ_g_*_(*Pe*)_ is the density of the gas in the diffusion chamber subjected to absorption (kg·m^−3^), *ρ*_(*DESs*)_ is the density of the *DES* (kg·m^−3^), and *w* is the mass of the *DES* in the diffusion chamber (*g*).

The relative molecular mass of the DES + water systems is calculated by Equation (7):

(7) $$M_{DES}=\frac{M_{HBA}\cdot n_{HBA}+M_{HBD}\cdot n_{HBD}+M_{H_{2}O}\cdot n_{H_{2}O}}{n}$$

where *n_HBA_* is the amount of *HBA*, *n_HBD_* is the amount of *HBD*, *M_H_*_2*O*_ is the relative molecular mass of water, *n_H_*_2*O*_ is the amount of water and *n* is the cumulative amount of the three substances.

**Figure 5:**
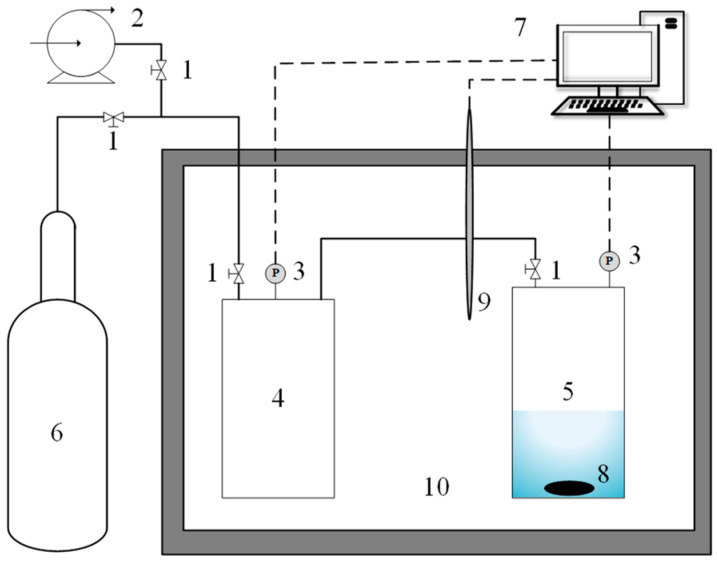
Schematic of the experimental setup used to determine gas solubility. 1: Needle valve; 2: Vacuum pump; 3: Pressure sensor; 4: Gas chamber; 5: Diffusion chamber; 6: CO_2_ cylinder; 7: Data Acquisition System; 8: Magnetic Rotor; 9: Temperature Sensor; 10: Constant Temperature Sink.

A gas solubility experimental system was built based on the isovolumetric saturation method. The solubility experimental system is shown in Figure 5. Its main components included: (1) piping system; (2) reaction chamber; (3) temperature control system; (4) vacuum pump; and (5) data acquisition system.

## 5. Conclusions

The impact of HBDs on the solubility of CO_2_ in a DES is more significant than that of HBAs due to the chemical reactions involved in the absorption process, where the amine reacts with CO_2_. When the HBDs are kept constant, the solubility of CO_2_ is found in the following descending order: 50 wt% TBAB/MEA > 50 wt% TBAB/MDEA, and 50 wt% TBPB/MEA > 50 wt% TBPB/MDEA. Similarly, when the HBAs are the same, the solubility of CO_2_ follows the descending order: 50 wt% TBAB/MEA > 50 wt% TBPB/MEA, and 50 wt% TBAB/MDEA > 50 wt% TBPB/MDEA.

As the temperature increases, the rate at which the DES system absorbs CO_2_ increases, while its solubility decreases slightly. At a certain temperature, increasing the proportion of the amine solution enhances the overall solubility of CO_2_ in the system. However, the formation of a more complex hydrogen-bonding structure within the DES can reduce the CO_2_ solubility of the MEA solution, which leads to a decreased utilization efficiency of MEA.

When comparing the CO_2_ solubility of the synthesized DES + water systems with the commonly used 30 wt% aqueous MEA solution, it is found that, under certain pressure ranges and at the same temperature, the CO_2_ solubility of 50 wt% TBAB/MEA (with a molar ratio of 1:10) and 50 wt% TBPB/MEA (with a molar ratio of 1:10) was higher than that of the 30 wt% aqueous MEA solution.

## Figures and Tables

**Table 1:** Fitting parameters.

Systems	A/(kg·m^−3^)	B/(kg·m^−3^·K^−1^)	R^2^
n_TBAB_:n_MEA_ = 1:8	1.24847	−7.28842 × 10^−4^	0.99905
n_TBAB_:n_MEA_ = 1:9	1.24701	−7.25945 × 10^−4^	0.99902
n_TBAB_:n_MEA_ = 1:10	1.24542	−7.22544 × 10^−4^	0.99895
n_TBAB_:n_MDEA_ = 1:8	1.26478	−7.43202 × 10^−4^	0.99867
n_TBAB_:n_MDEA_ = 1:9	1.26423	−7.41458 × 10^−4^	0.99860
n_TBAB_:n_MDEA_ = 1:10	1.26385	−7.39942 × 10^−4^	0.99855
n_TBPB_:n_MEA_ = 1:8	1.24991	−7.32167 × 10^−4^	0.99910
n_TBPB_:n_MEA_ = 1:9	1.24822	−7.28431 × 10^−4^	0.99904
n_TBPB_:n_MEA_ = 1:10	1.24550	−7.22521 × 10^−4^	0.99897
n_TBPB_:n_MDEA_ = 1:8	1.26578	−7.45715 × 10^−4^	0.99881
n_TBPB_:n_MDEA_ = 1:9	1.26427	−7.41711 × 10^−4^	0.99861
n_TBPB_:n_MDEA_ = 1:10	1.26472	−7.42246 × 10^−4^	0.99860

where A is a constant, and B represents the primary coefficient in the linear equation.

**Table 2:** Regression values of the solubility model parameters used in this paper.

Systems	a	b	c	d	e	The Maximum Relative Deviations %
n_TBAB_:n_MEA_ = 1:8	32.18	−15,420.36	8.31	74,352.19	−621.83	1.03
n_TBAB_:n_MEA_ = 1:9	50.40	−21,922.02	−82.46	109,660.46	−668.88	1.52
n_TBAB_:n_MEA_ = 1:10	28.69	−12,793.19	10.52	48,694.44	−344.33	1.48
n_TBAB_:n_MDEA_ = 1:8	62.24	−18,468.99	−318.51	95,012.65	283.98	1.73
n_TBAB_:n_MDEA_ = 1:9	−13.38	8478.80	143.74	−101,886.59	925.13	1.19
n_TBAB_:n_MDEA_ = 1:10	53.13	−15,273.12	−238.42	62,744.30	373.20	1.47
n_TBPB_:n_MEA_ = 1:8	0.14	−4032.65	213.57	−8744.48	−408.77	1.18
n_TBPB_:n_MEA_ = 1:9	12.03	−8203.25	140.83	17,699.03	−440.31	1.51
n_TBPB_:n_MEA_ = 1:10	27.49	−14,094.76	68.92	50,775.95	−532.78	1.04
n_TBPB_:n_MDEA_ = 1:8	59.90	−17,635.24	−316.34	93,432.50	291.29	2.06
n_TBPB_:n_MDEA_ = 1:9	27.99	−7174.00	−70.33	9439.45	362.81	1.98
n_TBPB_:n_MDEA_ = 1:10	40.94	−11,061.94	−184.92	42,846.74	382.71	1.51

**Table 3:** Various characteristics of the reagents used in the current work.

Molecular Formula	CAS	Producers	Mass Fraction Purity (Supplier)
C₁₆H₃₆BrN	1643-19-2	Aladdin	99.0%
C_16_H_36_PBr	3115-68-2	Aladdin	99.0%
C_2_H_7_NO	141-43-5	Aladdin	99.0%
C_5_H_13_NO_2_	105-59-9	Aladdin	99.0%

## Data Availability

Data available in a publicly accessible repository.

## Supplementary Materials

The following supporting information can be downloaded at: https://www.mdpi.com/article/10.3390/molecules29153579/s1, Figure S1: CO2 solubility curves of 50 wt% TBAB + MDEA and TBPB + MDEA eutectic solvents with pressure; Table S1: Experimental data of 50 wt% TBAB deep eutectic solvents density; Table S2: Experimental data of 50 wt% TBPB deep eutectic solvents density; Table S3: Solubility of CO2 in 50 wt% TBAB + MEA deep eutectic solvents; Table S4: Solubility of CO2 in 50 wt% TBAB + MDEA deep eutectic solvents; Table S5: Solubility of CO2 in 50 wt% TBPB + MEA deep eutectic solvents; Table S6: Solubility of CO2 in 50 wt% TBPB + MDEA deep eutectic solvents; Table S7: Tetrabutylammonium bromide-based DES + water systems; Table S8: Tetrabutylphosphonium bromide-based DES + water systems.
