# Supplementary material for: Deep Eutectic Solvent + Water System in Carbon Dioxide Absorption
Source: Molecules. 2024 Jul 29;29(15):3579. doi: 10.3390/molecules29153579 (PMC11314133; doi:10.3390/molecules29153579)
Supplement: Supplementary file 1 [file molecules-29-03579-s001.zip › molecules-3100237-supplementary.pdf]

Deep eutectic solvent + water system in carbon dioxide absorption

Jing Fan, Xin Zhang, Nan He, Fenhong Song，Hongwei Qu*

School of Energy and Power Engineering, Northeast Electric Power University, Jilin, Jilin 132012, People's Republic of China

***Corresponding author: quhongwei@neepu.edu.cn**

Table S1 Experimental data of 50wt% TBAB deep eutectic solvents density

***T*/(K)**	ρ_{exp}/(kg·m^{-3})					
	**DES1**	**DES2**	**DES3**	**DES4**	**DES5**	**DES6**
293.15	1.033913	1.033306	1.032662	1.045832	1.045751	1.045785
298.15	1.030663	1.030057	1.029472	1.042586	1.042549	1.042608
303.15	1.027333	1.026746	1.026188	1.039258	1.039237	1.039311
308.15	1.023952	1.023387	1.022847	1.035863	1.035855	1.035940
313.15	1.020512	1.019965	1.019446	1.032395	1.032399	1.032496
318.15	1.017013	1.016484	1.015985	1.028857	1.028873	1.028977
323.15	1.013457	1.012946	1.012462	1.025244	1.025272	1.025384
328.15	1.009844	1.009347	1.008882	1.021561	1.021598	1.021719
333.15	1.006171	1.005689	1.005239	1.017806	1.017851	1.017979
338.15	1.002438	1.001968	1.001533	1.013982	1.014033	1.014167
343.15	0.998646	0.998187	0.997767	1.010086	1.010141	1.010281
348.15	0.994794	0.994347	0.993939	1.006121	1.006178	1.006321
353.15	0.990890	0.990453	0.990056	1.002091	1.002150	1.002293
358.15	0.986925	0.986500	0.986109	0.997993	0.998052	0.998199
363.15	0.982903	0.982487	0.982101	0.993831	0.993888	0.994038

Table S2 Experimental data of 50wt% TBPB deep eutectic solvents density

***T*/(K)**	ρ_{exp}/(kg·m^{-3})					
	**DES7**	**DES8**	**DES9**	**DES10**	**DES11**	**DES12**
293.15	1.034415	1.033780	1.032767	1.046159	1.045736	1.046011
298.15	1.031129	1.030540	1.029562	1.042901	1.042507	1.042792
303.15	1.027773	1.027217	1.026278	1.039553	1.039199	1.039480
308.15	1.024366	1.023837	1.022937	1.036138	1.035817	1.036096
313.15	1.020904	1.020399	1.019535	1.032651	1.032361	1.032637
318.15	1.017388	1.016905	1.016074	1.029095	1.028833	1.029106
323.15	1.013815	1.013350	1.012551	1.025467	1.025231	1.025501
328.15	1.010187	1.009737	1.008969	1.021771	1.021556	1.021824
333.15	1.0065	1.006065	1.005325	1.018001	1.017807	1.018073
338.15	1.002756	1.002336	1.001623	1.014162	1.013988	1.014250

343.15	0.998951	0.998545	0.997859	1.010256	1.010097	1.010355
348.15	0.995088	0.994697	0.994036	1.006280	1.006136	1.006392
353.15	0.99117	0.990794	0.990153	1.002239	1.002107	1.002359
358.15	0.987191	0.986826	0.986204	0.998126	0.998006	0.998254
363.15	0.983152	0.982800	0.982196	0.993945	0.993836	0.994081

TableS3 Solubility of CO_2 in 50 wt% TBAB+MEA deep eutectic solvents

P (kPa)	x (g·g^{-1})	P (kPa)	x (g·g^{-1})	P (kPa)	x (g·g^{-1})
**50wt%DESs（n_{TBAB}:n_{MEA}=1:8）**					
T=303.15K		T=313.15K		T=323.15K	
129.3	0.143	132.5	0.136	131.3	0.130
263.5	0.152	260.8	0.146	278.0	0.139
399.3	0.160	372.5	0.153	401.5	0.146
533.8	0.166	503.3	0.159	515.8	0.152
652.5	0.171	627.0	0.164	643.3	0.158
762.0	0.175	758.8	0.168	778.6	0.162
895.3	0.178	918.3	0.173	922.0	0.168
**50wt%DESs（n_{TBAB}:n_{MEA}=1:9）**					
T=303.15K		T=313.15K		T=323.15K	
115.0	0.148	124.5	0.141	120.5	0.132
258.3	0.158	240.8	0.149	259.8	0.141
362.8	0.164	370.0	0.157	401.5	0.149
528.5	0.171	531.3	0.163	523.0	0.155
618.5	0.174	648.5	0.167	651.5	0.160
752.8	0.179	773.0	0.172	786.3	0.163
901.0	0.183	890.3	0.175	903.8	0.166
**50wt%DESs（n_{TBAB}:n_{MEA}=1:10）**					
T=303.15K		T=313.15K		T=323.15K	
108.0	0.154	122.5	0.147	108.5	0.137
248.5	0.167	243.8	0.159	249.8	0.149
367.3	0.175	390.5	0.169	410.0	0.158
517.8	0.182	509.0	0.174	532.3	0.164
702.8	0.190	631.3	0.178	651.3	0.169
827.5	0.194	748.8	0.181	754.8	0.172
916.3	0.196	887.5	0.185	886.5	0.174

TableS4 Solubility of CO_2 in 50 wt% TBAB+MDEA deep eutectic solvents

P (kPa)	*x* (g·g^{-1})	*P* (kPa)	*x* (g·g^{-1})	*P* (kPa)	*x* (g·g^{-1})
		**50wt%DESs（n_{TBAB}:n_{MDEA}=1:8）**			
T=303.15K		*T*=313.15K		*T*=323.15K	
113.0	0.118	149.8	0.108	192.3	0.095
265.0	0.133	238.3	0.118	249.8	0.102
407.8	0.139	358.8	0.127	351.3	0.111
548.3	0.142	518.0	0.135	561.5	0.122
693.0	0.146	638.5	0.138	669.8	0.125
857.8	0.149	752.3	0.141	774.0	0.128
973.0	0.151	893.5	0.143	901.5	0.133
		**50wt%DESs（n_{TBAB}:n_{MDEA}=1:9）**			
T=303.15K		*T*=313.15K		*T*=323.15K	
125.0	0.124	163.8	0.115	131.5	0.104
278.3	0.136	249.0	0.123	199.8	0.111
401.3	0.142	369.5	0.131	359.5	0.124
553.8	0.148	541.3	0.139	524.0	0.131
719.0	0.152	664.5	0.143	664.8	0.135
876.8	0.154	780.8	0.146	781.3	0.138
1025.8	0.156	898.5	0.148	892.5	0.141
		**50wt%DESs（n_{TBAB}:n_{MDEA}=1:10）**			
T=303.15K		*T*=313.15K		*T*=323.15K	
126.0	0.128	139.5	0.116	159.8	0.104
265.5	0.139	285.8	0.128	228.3	0.109
396.3	0.145	441.3	0.135	346.5	0.118
496.0	0.149	564.0	0.140	502.3	0.126
645.8	0.151	665.8	0.142	648.8	0.131
785.3	0.153	825.3	0.146	795.3	0.136
989.0	0.156	948.0	0.148	930.0	0.139

Table S5 Solubility of CO_2 in 50 wt% TBPB+MEA deep eutectic solvents

P (kPa)	x (g·g^{-1})	P (kPa)	x (g·g^{-1})	P (kPa)	x (g·g^{-1})
		**50wt%DESs（n_{TBPB}:n_{MEA}=1:8）**			
T=303.15K		T=313.15K		T=323.15K	
113.5	0.141	130.5	0.135	133.3	0.129
229.8	0.152	257.8	0.144	276.0	0.138
339.0	0.159	368.3	0.149	397.8	0.143
494.0	0.166	499.5	0.154	513.5	0.147
613.3	0.170	621.0	0.158	641.8	0.151
800.3	0.176	755.8	0.162	774.3	0.154
950.3	0.183	914.5	0.166	902.0	0.157
		**50wt%DESs（n_{TBPB}:n_{MEA}=1:9）**			
T=303.15K		T=313.15K		T=323.15K	
114.5	0.147	128.3	0.140	116.3	0.132
262.3	0.160	238.3	0.149	262.5	0.142
362.0	0.166	374.5	0.156	398.3	0.148
489.5	0.172	529.8	0.162	525.5	0.153
618.8	0.177	651.0	0.166	656.5	0.157
761.5	0.182	777.8	0.170	786.8	0.160
895.8	0.185	892.5	0.173	905.0	0.163
		**50wt%DESs（n_{TBPB}:n_{MEA}=1:10）**			
T=303.15K		T=313.15K		T=323.15K	
111.3	0.153	119.3	0.145	104.5	0.135
222.5	0.163	245.8	0.155	252.8	0.146
360.0	0.172	386.3	0.163	407.3	0.153
572.8	0.182	514.0	0.168	535.5	0.158
688.5	0.186	626.3	0.172	648.3	0.161
795.8	0.190	752.8	0.176	755.5	0.164
915.3	0.193	891.5	0.180	884.8	0.167

TableS6 Solubility of CO_2 in 50 wt% TBPB+MDEA deep eutectic solvents

P (kPa)	x ($g \cdot g^{-1}$)	P (kPa)	x ($g \cdot g^{-1}$)	P (kPa)	x ($g \cdot g^{-1}$)
**50wt%DESs（n_{TBPB}:n_{MDEA}=1:8）**					
T=303.15K		T=313.15K		T=323.15K	
104.8	0.115	153.3	0.106	189.8	0.092
284.5	0.131	234.5	0.117	252.3	0.101
381.0	0.136	362.5	0.126	346.0	0.110
503.5	0.139	521.5	0.133	558.8	0.121
641.3	0.142	641.0	0.136	671.3	0.125
763.5	0.144	759.5	0.138	776.8	0.128
907.8	0.146	889.0	0.141	896.5	0.131
**50wt%DESs（n_{TBPB}:n_{MDEA}=1:9）**					
T=303.15K		T=313.15K		T=323.15K	
115.5	0.119	165.3	0.109	127.8	0.086
246.8	0.135	253.3	0.120	203.0	0.098
404.3	0.142	371.8	0.128	361.5	0.115
526.3	0.146	538.8	0.134	520.0	0.124
641.5	0.148	662.0	0.138	659.3	0.130
778.0	0.150	783.8	0.141	773.8	0.133
915.8	0.152	900.3	0.143	888.5	0.135
**50wt%DESs（n_{TBPB}:n_{MDEA}=1:10）**					
T=303.15K		T=313.15K		T=323.15K	
119.3	0.121	142.8	0.109	154.5	0.090
239.8	0.135	280.3	0.127	234.3	0.105
396.5	0.143	438.3	0.135	342.5	0.116
559.3	0.148	556.0	0.140	497.5	0.126
674.0	0.151	670.0	0.143	637.5	0.132
795.0	0.153	804.8	0.145	785.0	0.136
917.3	0.155	938.5	0.147	929.8	0.139

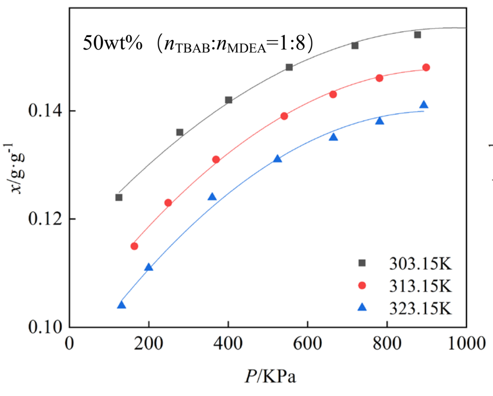

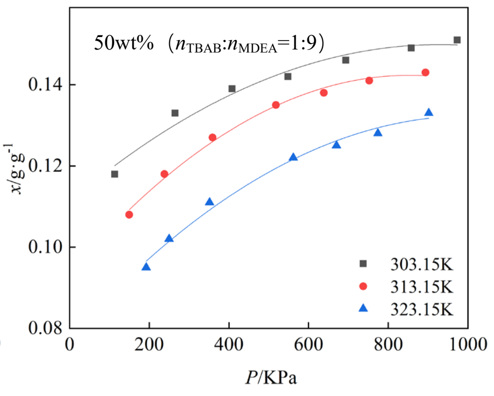

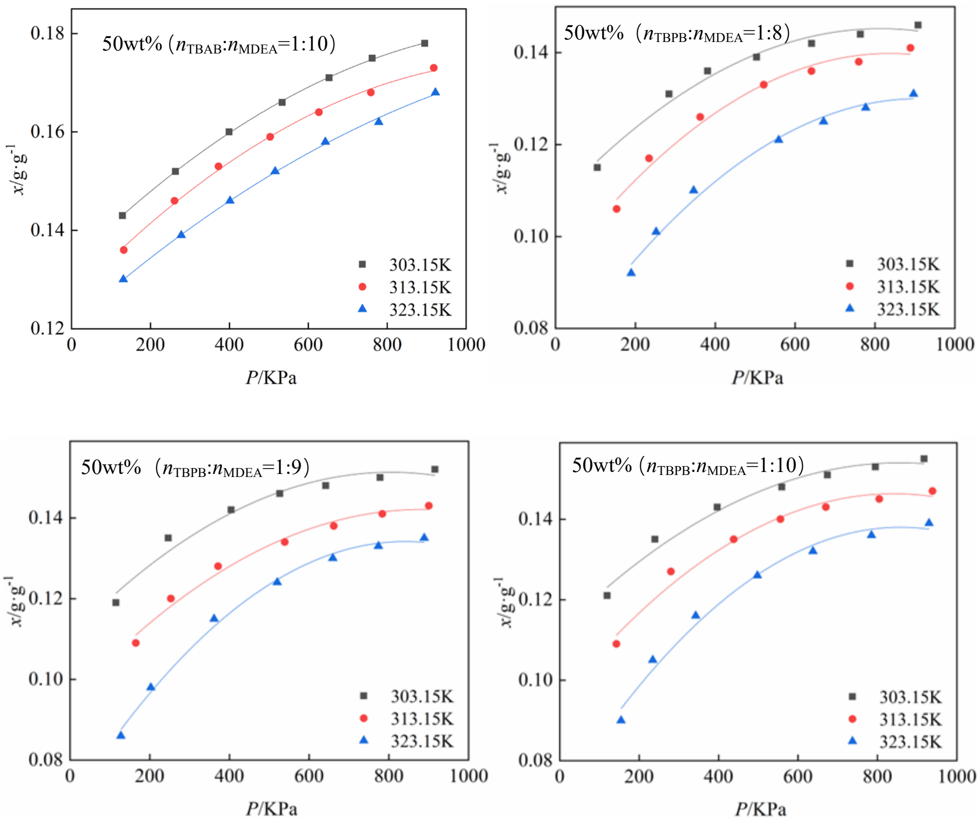

CO_2 solubility curves of 50wt%TBAB+MDEA and TBPB+MDEA eutectic solvents with pressure.

Table S7. Tetrabutylammonium bromide-based DES + water systems

HBA	HBD	Molar ratio	Solutions	Moisture content/wt %
Tetrabutylammonium bromide	Ethanolamine	1：8	DES1	49.83
		1：9	DES2	49.75
		1：10	DES3	49.78
	N-methyldiethanolamine	1：8	DES4	49.91
		1：9	DES5	49.90
		1：10	DES6	49.84

Table S8. Tetrabutylphosphonium bromide-based DES + water systems

HBA	HBD	Molar ratio	Solutions	Moisture content/wt %
Tetrabutylphosphonium bromide	Ethanolamine	1：8	DES7	49.78
		1：9	DES8	49.76
		1：10	DES9	49.74
	N-methyldiethanolamine	1：8	DES10	49.73
		1：9	DES11	49.71
		1：10	DES12	49.89
